# Farming without Glyphosate?

**DOI:** 10.3390/plants9010096

**Published:** 2020-01-11

**Authors:** Hugh J. Beckie, Ken C. Flower, Michael B. Ashworth

**Affiliations:** Australian Herbicide Resistance Initiative (AHRI), UWA School of Agriculture and Environment, University of Western Australia, Perth WA 6009, Australia; ken.flower@uwa.edu.au (K.C.F.); mike.ashworth@uwa.edu.au (M.B.A.)

**Keywords:** glyphosate ban, herbicide resistance, integrated weed management, maximum residue level, social license

## Abstract

Recent statements from scientific organisations and court decisions have resulted in widespread public interest and concern over the safety of glyphosate, the most popular and effective herbicide used worldwide. Consequently, glyphosate-based products are under intense scrutiny from governments at all levels. Some jurisdictions have already banned or restricted its use, which will adversely impact international trade in bulk grain commmodities if glyphosate residues are detected. The possibility of farming without glyphosate is becoming an important issue facing the agri-food research and development sector. Contingency plans need to be formulated if that scenario becomes a reality. In this review, we briefly summarize international events that have led to this possible situation, describe current glyphosate usage in major agronomic field crops worldwide, outline possible alternatives to glyphosate in two agroregions and perform bioeconomic model scenarios of southern Australian broadacre cropping systems without the herbicide. Model predictions suggest that we can farm profitably without glyphosate by consistently utilizing key non-herbicidal weed management practices combined with robust pre-emergence soil residual herbicide treatments. However, maintaining low weed seed banks will be challenging. If the social license to use glyphosate is revoked, what other pesticides will soon follow?

## 1. Introduction

Glyphosate, a non-selective herbicide with a unique site of action (SOA), was introduced in 1974 by Monsanto (Roundup™) for use in agriculture and for industrial or ruderal (non-crop disturbed) vegetation control. The herbicide is commonly used in fallow fields, orchards, vineyards, along fencelines, etc., for bare ground weed control [1]. In non-glyphosate-resistant (GR) crop fields, it is used mainly as a burndown, non-residual weed control treatment applied before crop seeding or crop seedling emergence (PRE). With little to nil soil residual activity, there are no re-cropping restrictions following its application before seeding. Glyphosate greatly facilitated the adoption of minimum or no-tillage cropping in the late 1970s and 1980s, which enabled greater yields and yield stability especially in arid to semiarid regions through soil moisture conservation and more timely crop establishment [2]. Most importantly, it mitigated the rapid decline in soil quality due to excessive tillage that was prevalent in many global agroregions up until the 1980s. Glyphosate is also commonly used pre-harvest in cereals and annual legumes, mainly targeting hard-to-control annual or perennial weed species or as a desiccant to hasten uniform crop maturity. With the advent of GR (Roundup Ready™) crop cultivars beginning in 1996, the herbicide could now be applied multiple times post-emergence (POST) in-crop to obtain an unsurpassed level of weed control. Roundup™ patent expiration in 2000 was followed by even greater use of the herbicide active ingredient, with generic products of differing formulations now manufactured by numerous companies. Today, it is by far the most widely used herbicide globally [3]. 

In an assessment of weed resistance risk, glyphosate was rated as low risk when applied to a field ≤20 times in total [4]; recently it was rated at moderate risk overall, but high risk for some resistance-prone weed species such as *Amaranthus* and *Lolium* spp. [5]. Even with the evolution of GR weed populations in crop and non-crop areas, first documented in 1996 and now totalling 45 species [6], glyphosate usage has not declined because of its low cost and broad-spectrum weed efficacy. However, the continued evolution and spread of GR weeds is expected to eventually lead to decreased glyphosate use [7]. Restrictions on glyphosate use have already been mandated in some food crop production contracts, such as milling oats (*Avena sativa* L.) [8]. There will be increasing demand from buyers for no pre-harvest glyphosate use because of concerns over residue in the harvested grain. 

Environmental concerns have been raised because of widespread detection of glyphosate residues in water and soil, persistence and off-target movement [9]. Together with human health concerns, the predicted future decline in glyphosate use may quickly accelerate. Unexpected ‘storm clouds’ appeared in 2015, when glyphosate was classified as a ‘probable carcinogen’ by the World Heath Organization International Agency for Research on Cancer [10]. This designation resulted after a hazard-based assessment, which was followed by court cases in 2019 in California, United States (U.S.) that decided in the plaintiffs’ favour. Collectively, these decisions have resulted in widespread public interest and concern over the safety of glyphosate or glyphosate-based products. On the other hand, a 2018 agricultural health study by the U.S. National Cancer Institute found no association between glyphosate-based herbicides and cancer [11]. Moreover, pesticide regulators, notably the U.S. Environmental Protection Agency (EPA) and European Food Safety Authority (EFSA), maintain that glyphosate-based herbicides are not likely to be carcinogenic and that the risk (hazard × exposure) of using glyphosate is acceptable when used as labelled. In the past, mitigation measures to lower human exposure or environmental risks were sometimes imposed as a condition of pesticide registration or usage.

Nevertheless, the publication of three studies in reputable scientific journals, the first in 2016 [12] followed by two others in 2019 [13,14], may mark the beginning of the end for this ‘once in a century’ herbicide [3]. All three studies determined a link between glyphosate exposure and non-Hodkin lymphoma. These published scientific journal articles (and others that may follow) may indeed accelerate or precipitate the restriction or outright ban of the herbicide in numerous countries or jurisdictions, such as planned or occurring in Austria, France, Germany and Vietnam. If or when that happens, international trade in many agricultural bulk commodities will likely be impacted because of the lack of maximum residue levels (MRLs). These thresholds are set only for pesticides that are registered in the importing country. Therefore, grain shipments could be turned back to country of origin if residues of unregistered pesticides are detected [15]. 

How much longer will glyphosate play a major role in weed control [16]? Around the world, those involved in the agri-food research and development sector are proactively planning and preparing for a worst-case scenario of a glyphosate-free agriculture. The existing social license allowing the unhindered use of glyphosate in agricultural and non-agricultural settings may soon be restricted or revoked by different levels of government in countries around the world. In developed countries, most voters live in urban centres; politicians naturally cater to eligible voters of whom farmers are becoming a smaller and smaller percentage over time (usually <5% nationally). 

A workshop of weed scientists or practitioners and agronomists from across Australia was held in Sydney in November 2019, to contemplate and discuss various scenarios of farming in a herbicide-limited environment (see Acknowledgments section). The five scenarios examined were the following: (1) no knockdowns (burndown treatments), (2) no PRE herbicides, (3) no in-crop or POST herbicides, (4) no pre-harvest herbicide treatments and (5) no herbicides entirely. We believe that the probability of scenarios 1 and 4 being realized within five years is the greatest because of threat of losing key non-selective herbicides in these two application windows—glyphosate, paraquat and diquat.

In the following section, we briefly describe glyphosate usage globally from 1974 to present and highlight how the herbicide has changed the way major agronomic crops are produced in the Americas, Europe and Asia-Oceania region. We then describe possible alternatives to glyphosate in southern Australian grain cropping systems, which is followed by case study scenarios of farming without glyphosate using a bioeconomic simulation model. Lastly, we suggest some future directions for research, development and extension for the possible transitioning from farming with glyphosate to farming without glyphosate or key herbicides in general.

## 2. Glyphosate Usage: 1974 to Present

Globally, 90% of glyphosate is applied to agricultural land and 10% to non-agricultural land (ruderal, industrial or urban areas); between 1974 and 2014, 8.6 billion kg of glyphosate active ingredient (ai) was applied (Table 1; [17]). Of the 90% of glyphosate applied to agricultural land, over half (56%) was applied to GR crops. Since the first introduction of GR crop cultivars in 1996, glyphosate use has risen 15-fold [17]. Glyphosate and GR crops have revolutionized weed management in the Americas. The positive agronomic, economic and environmental benefits of glyphosate and GR crops have been extensively reviewed e.g., in [3,18,19], such as reduced energy or herbicide costs, simplified and better weed management, improved soil health through reduced tillage and lower environmental impact overall. However, the rising incidence of GR weeds since 1996 is rapidly decreasing the value of glyphosate and GR crop cultivars [20]. Many growers have lost the benefit of being able to use less herbicide because of GR weeds; for example, U.S. soybean (*Glycine max* (L.) Merr.) growers now use 28% or 0.30 kg ha^−1^ more herbicides [21]. Cultivars with an alternative herbicide-resistant (HR) trait (e.g., glufosinate, dicamba) or combined (stacked) HR traits, are rapidly being adopted as a means to manage weed populations resistant to glyphosate or multiple SOA herbicides [22].

### 2.1. The Americas

Arable dryland agriculture in South America is mainly concentrated in Brazil and Argentina. The high level of adoption of no-tillage and GR crops, mainly soybean, in South America has greatly increased the use of glyphosate as the primary tool to control weeds [23]. Brazil, with nearly half of the continent’s cultivated land area, planted over half of that area (37 million ha) to mostly GR soybean in 2019/20 [24]. With glyphosate as the sole herbicide applied multiple times during the crop growing season (PRE and POST), selection pressure for GR weeds is intense. Glyphosate is often applied at least three to five times a year to fields where GR soybean is grown [25]. Of the presently documented eight GR weed species in Brazil, all were selected in GR soybean. Some of the GR species were also selected in GR maize (*Zea mays* L.), wheat (*Triticum aestivum* L.) (PRE or pre-harvest application) or fruit orchards [6]. 

Since 1974 in the U.S., over 1.6 billion kg of glyphosate ai have been applied, or 19% of global glyphosate use (8.6 billion kg ai) [17]. Over 70% cumulative glyphosate use from 1974 to 2014 occurred from 2005 to 2014 (6.1 billion kg ai). Similar to the global proportion, 90% of glyphosate (113.4 million kg ai) was applied to agricultural land in the U.S. in 2014 (Table 2). Soybean and maize accounted for 77% of glyphosate applied to agricultural land, with >90% being GR cultivars. The average number of glyphosate applications per year in GR maize, soybean and cotton (*Gossypium hirsutum* L.) fields in the U.S. were 1.38, 2.03, and 3.29, respectively [26]. In the U.S. in 2015, glyphosate accounted for 26% of maize, 43% of soybean, and 45% of cotton herbicide applications [27]. Since the first report (2001) of a GR weed selected in a GR crop in the U.S. [28], a majority of the other U.S.-documented GR weeds since then have been selected in GR crops [29]. Due to the widespread incidence of glyphosate resistance in key driver weeds of U.S. maize, soybean and cotton, such as *Amaranthus* spp., it has been stated that glyphosate is already lost to the U.S. [30]. Thus, the loss of glyphosate today in the Americas would have a much less adverse impact on agronomic crop production than 20 years ago. A similar situation occurs in eastern Canada where maize and soybean cropping dominates. However, cultivation of GR canola (*Brassica napus* L.) in western Canada, which comprises about 40% of the crop area, has not significantly selected for GR weeds because of the dominance of glufosinate-resistant cultivars and crop rotation diversity [31]. The loss of pre-harvest glyphosate would adversely impact perennial weed control in pulse crops in North America as there are no good alternative herbicides.

### 2.2. Europe

Although European cropping systems are diverse, crop rotations are dominated by winter crops—wheat, barley (*Hordeum vulgare* L.), oilseed rape/canola—as well as maize or other summer crops depending on the climatic zone. Europe is the largest producer of wheat (soft wheat mainly in Central Europe; durum wheat in Italy and Spain), with France, Germany, Poland and the United Kingdom producing more than half of the region’s small-grain cereals. Compared with other global regions, tillage is still prevalent, although minimum tillage is expanding slowly [32]. As GR crops are not widely grown in the European Union, glyphosate is mainly used PRE, pre-harvest or post-harvest in crop rotational phases or in fallowed fields. Weed species that have evolved resistance to glyphosate include *Conyza* spp. in perennial crops (mainly olive and citrus groves in the Mediterranean areas) and *Lolium* spp. in vineyards and to a lesser extent in wheat, where the use of glyphosate after harvest and before sowing is high [33]. 

### 2.3. Asia-Oceania

Agriculture in Asia-Oceania is very diverse in the number of crops grown and climatic zones across the region. Rice (*Oryza sativa* L.) is the dominant crop, followed by wheat, maize, palm oil, and natural rubber. Compared with other global regions, glyphosate use is much less prevalent. Annual glyphosate use (million kg ai) in seven countries in the region are the following: Australia, 24.1; China, 20.1; Thailand, 15.3; India, 14.2; Indonesia, 9.7; Vietnam, 3.2; Philippines, 2.1 [34]. Glyphosate use as a percentage of total herbicide use (in terms of ai and spray area, respectively), is the following: Australia, 32/17; China, 13/7, Thailand, 33/19; India, 37/24; Indonesia, 73/35; Vietnam, 36/35; Philippines 48/38; in Australia, glyphosate use is greatest in cotton, cereal and canola cropping systems, as well as vineyards [34]. In palm oil and rubber plantations in Indonesia and Malaysia, GR weeds such as goosegrass (*Eleusine indica* (L.) Gaertn.) have been selected [6]. In Australia, there are 17 GR weed species, selected in crop fields, vineyards or bareground areas (e.g., along fencelines). GR weed species are more abundant in South Australia and New South Wales than in Western Australia. Most GR populations were not selected in GR crops as GR canola has only been cultivated relatively recently (since 2008 in New South Wales and Victoria and since 2010 in Western Australia). Although the cultivation of GR canola is increasing, most canola grown is triazine-resistant (TR). GR cotton is grown in southeastern Australia; mandated rigorous stewardship practices has prevented significant selection of GR weeds [35]. 

## 3. Alternatives to Glyphosate 

To date, there are few published reports on the impact of farming without glyphosate. Alternatives to glyphosate in two agricultural regions are described in this section. An overview of alternatives in France are initially discussed, followed by a more detailed assessment of southern Australian grain cropping systems. 

**Figure 1:**
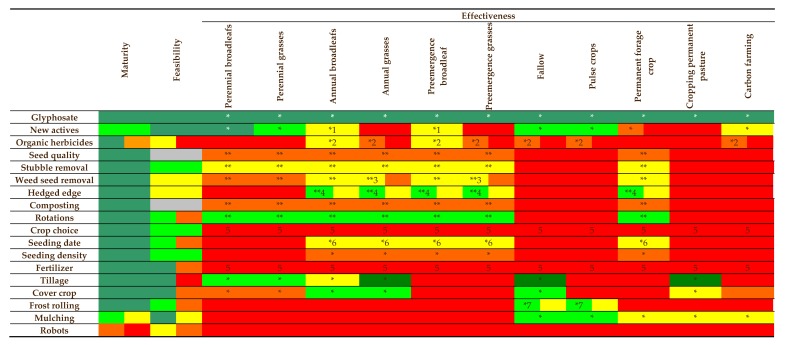
The potential of different methods to control weeds in agronomic crops in France (adapted from Reboud et al. [36]). Each method is characterized by its level of technological maturity, ease of implementation (feasibility) and efficacy. The following colour codes are used: level of technological maturity—dark green: already marketed or used; light green: proven effective in many cases; orange: method validated under specific experimental conditions; yellow: proof of concept provided, active research phase; red: lower level, basic principles only formalized. Feasibility and efficiency—dark green: very high; light green: high; orange: medium; yellow: poor; red: very poor (colour codes of the cells divided in two indicate the extreme classes that frame the variability of the criterion concerned (grey: not relevant). Ratings 1 to 7: (1) only the combination of multiple active substances would provide sufficient broadspectrum weed control; (2) economic constraints ($300 euros ha^−1^) and logistics (12 to 16 L ha^−1^); (3) not effective on foxtail spp.; (4) only works on certain floras; (5) suitable for herbicide reduction; (6) easier and more effective in spring cultivation and cereals vs. rapeseed; (7) dependent on availability the number of frost days (‘*’: efficiency varies by year; ‘**’: long-term maintenance of low weed seed banks).

A report was prepared by the French government in 2017 to assess the impact of a partial or total glyphosate ban in France by 2021 [36]. In 2016, 9.1 million kg ai glyphosate was used in France, with 16% of that in non-agricultural areas. The majority of glyphosate usage in agricultural fields occurs before crop seeding. Glyphosate is most important for cereal and oilseed crop production. If or when glyphosate is severely restricted or banned largely because of environmental concerns of residues in water and soil, proposed alternative means of weed management will rely on physical or mechanical methods (tillage, cutting, etc.), cultural tools (seeding date and rate, cover crops, etc.), as well as use of alternative herbicides (Figure 1). Some of these alternative methods of weed control are envisioned as being applied in a site-specific manner. They recommend research and development around precision agricultural systems, mechanical weed control systems, bioherbicides and new crop and cover crops and varieties.

In Australian cropping systems, weeds cost growers USD 2.3 billion annually, which includes USD 507 million in lost yield revenue with the remainder in additional weed control costs [37]. Of these costs, about 20% were estimated to come from fallow herbicide costs, 62% from in-season herbicide applications, and the remaining 18% from non-herbicide costs. No-tillage is widely adopted on large-scale cropping and mixed farms in Australia [38,39]. This high adoption is due to the soil and water conservation benefits as well as yield improvements from earlier and more reliable crop establishment. The uptake of no-tillage in regions like Western Australia was largely driven by farmers in response to wind erosion that was caused by tillage and minimal soil cover. Consequently, stubble retention is a key component of the no-tillage system. 

To be sustainable, alternatives to glyphosate must be considered in the context of the no-tillage system and stubble retention. In Australia, most glyphosate is used before crop seeding and to maintain summer and winter fallows, although increasing amounts are being used in GR cotton and canola crops. This section reviews options for cropping without glyphosate in fallow/pre-seeding, in-crop situations and for GR crops.

### 3.1. Fallow/Pre-Seeding

Effective fallow weed control is considered crucial to maintain low weed seed banks, prevent a ‘green bridge’ that acts as a vector for pests and disease incursions and conserve soil water for the subsequent crop. As in many countries with limited GR crops, the vast majority of glyphosate use on Australian grain-growing farms occurs before seeding (in fallow). Glyphosate is used alone or in a mixture with other herbicides to improve its efficacy on dicotyledon weed species such as those from the Brassicaceae, Asteraceae and Fabaceae families. As part of a strategy to fight herbicide resistance, glyphosate is also recommended as a ‘double knockdown’ with paraquat/diquat applied as a second knockdown herbicide.

Use of remote or proximal sensing to detect weed patches will have an important role in fallow weed management in the absence of glyphosate. Such real-time sensors, which can discriminate plants from soil, are already used for precision weed control in fallow fields in Australia [22,40]. These optical sprayers could be used to apply alternative weed-specific active ingredients. Alternative herbicides may include paraquat/diquat, although they will have limited impact on more established and perennial weeds. Use of POST grass herbicides are unlikely to be of use because of widespread weed resistance across many of the grain-cropping areas of Australia. Alternatively, these sensors have been attached to rapid-response, hydraulically controlled tynes with wide sweeps for shallow ‘targeted tillage’ [41]. This technology is likely to become more widespread in Australian cropping systems without glyphosate. As a variation of this, images taken from satellite or an unmanned aerial vehicle (UAV) could be used to map weed patches, followed by site-specific tillage (without the specialised tynes) and/or spraying.

Controlling weed seed set in pastures is likely to become more important, as weed-free legume pastures would benefit both the livestock and cropping enterprise. Farmers who have livestock can heavily graze pastures to prevent the weeds setting seed. Legume pastures such as *Biserrula pelecinus* could prove useful for ‘targeted grazing’, as the legume is unpalatable at certain growth stages and livestock preferentially graze the weeds [42].

Strategic use of mouldboard ploughing, about every 10 years, may also be considered to reset the weed seed bank to a low level, particularly if combined with other soil amelioration activities like lime incorporation or burying water-repellent sand [43]. Shallow tillage (0–5 cm), potentially in the form of rod-weeding, may be useful to remove weeds. However, more research would be required to determine the effect of widespread tillage on seed bank dynamics, soil water and particularly organic carbon levels, which are concentrated in the top 10 cm in many of these soils [44]. Such shallow tillage should also leave sufficient crop residue on the soil surface. The impact of a disturbed soil surface on efficacy of no-tillage seeding machinery and subsequent crop establishment would need to be investigated. 

### 3.2. In-Crop

Wheat is the most important grain crop in Australia, followed by barley, canola, cotton and lupin (*Lupinus* spp.). A major trend over the past decade has been ‘dry’ seeding. Twenty years ago, farmers typically waited for a significant rain event before seeding in May or June. However, more erratic rainfall patterns, larger farm sizes over time (now averaging ≥4000 ha in Western Australia) and loss of grain yield potential with delayed seeding has resulted in earlier seeding typically commencing in April or early May. To enable early seeding, it is especially important that weeds be well controlled in the preceding fallow period as noted above. 

Like in fallow, use of weed-sensing systems in-crop is likely to play an increasingly important role in weed management. Systems that detect weed patches or individual weeds have been studied, including spectral, fluorescence, photogrammetry/3D and laser imaging [40]. The mapping of weed patches early or late in the growing season could be a powerful tool for targeted weed control, as well as allowing farmers to determine the long-term effectiveness of different control measures. A weed map from the past growing season that is used to predict the following season weed threat would allow farmers to ‘stack’ a number of targeted control measures, both chemical and non-chemical, thereby ensuring weed control diversity. For example, identified weed patches could have higher rates and/or more diverse mixtures of PRE herbicides, very high crop seeding rates (even broadcast in the patches), inter-row tillage and targeted POST herbicide applications and reduced harvest height to ensure weed seed interception for harvest weed seed control (HWSC). As part of this patch-intensive management, more expensive herbicides may be justified because of the reduced area being treated. Pre-harvest site-specific herbicide application could reduce levels of herbicide residues detected in harvested crop seedlots.

With reduced glyphosate use, there is likely to be even greater reliance on PRE herbicides, particularly those which are not readily degraded by soil microbes. This reliance will increase selection pressure of these soil residual herbicides for resistance. Therefore, they will need to be managed carefully through a diversity of weed control tactics, including crop rotation that facilitates the use of PRE herbicides with different SOA as well as other weed control measures. Soil residual PRE herbicides such as trifluralin, triallate, atrazine, prosulfocarb or pyroxasulfone are commonly applied just before or at seeding to control key weeds, such as annual ryegrass (*Lolium rigidum* Gaud.) wild radish (*Raphanus raphanistrum* L.) and bromegrass (*Bromus* spp.). There is less reliance on POST herbicides in cereal crops because of widespread herbicide resistance, especially to acetyl-CoA carboxylase or acetolactate synthase-inhibiting herbicides. Crop-topping with paraquat/diquat, especially in pulse crops, can be performed to control weed seed set of escapes later in the growing season (pre-harvest), or to desiccate the crop for even maturity to facilitate direct-combining. Crop-topping is especially valuable in controlling weed species such as *Bromus* spp. that shed a large proportion of their seed prior to harvest. If the markets are available, hay cutting may become more important because of its weed control efficacy, with the crop and weed biomass cut and removed before the weeds mature. The effect of greater nutrient and biomass removal in the hay on soil fertility and organic carbon will have to be carefully managed. Finally, HWSC has been demonstrated to reduce and maintain low levels of weed populations [45]. Therefore, this tool will be an important part of any integrated weed management (IWM) system without glyphosate. However, weed populations will need to be monitored as the effectiveness and recurrent use of HWSC may select for biotypes that avoid seed capture at crop harvest [46]. 

In summary, a scenario of no knockdown (PRE) or pre-harvest (e.g., crop-topping) glyphosate treatments permitted in grain crops in southern Australia (which may include paraquat/diquat [15]) may result in the following consequences or alternative weed management practices, tactics or strategies [47]: (1) accelerated trend towards dry or early seeding, placing increased reliance on PRE soil residual herbicides and crop competition; (2) greater integration of pasture phases with or without livestock grazing in the cropping rotation; (3) more plus earlier hay cutting (cutting the crop for fodder production before maturity for weed seed set control); (4) greater attention to controlling weeds in fallow with selective PRE or POST herbicides or strategic tillage; (5) less production of inherently poor weed-competitive pulse crops, with greater attention to late season weed control via weed wiping or clipping above the crop canopy; (6) move to wider broadleaf crop rows and inter-row tillage or shielded spraying; and (7) greater use of PRE or early POST mechanical weed control, such as harrowing, rod-weeding or rotary hoeing. 

### 3.3. GR Crops

Glyphosate and GR crops have improved weed management in cotton and canola crops in Australia. Since its release in Australia in 2001, glyphosate use in GR cotton (99% of crop area) has replaced a complex range of herbicide SOA treatments required to control the diverse array of weeds [48]. The introduction of GR cotton cultivars provided a number of benefits for weed management, including (1) reduced dependence on residual herbicides, (2) improved control of some of the more difficult-to-control weeds, (3) greater flexibility in weed management programs, (4) reduced chipping and tillage expenses, and (5) improved establishment and vigour of young cotton seedlings by reducing the use of PRE residual herbicides. However, as a result of glyphosate overreliance, glyphosate resistance has evolved in populations of annual sowthistle (*Sonchus oleraceus* L.), flaxleaf fleabane (*Conyza bonariesis* (L.) Cronq.) and awnless barnyardgrass (*Echinochloa colona* (L.) Link). The first commercial release of GR canola in Australia was in 2008. GR hybrid cultivars yield 18% more than TR open-pollinated (OP) cultivars, and 10% more than TR hybrids [49]. Despite the lower yield potential, TR OP cultivars are predominantly grown, occupying almost 80% of the Western Australia canola area (with only 2% TR hybrid cultivars). GR cultivars comprise 18% of crop area, with only 1% of area planted to imidazolinone-resistant canola [49]. Seed costs and expected yields are important determinants in varietal selection by growers. The GR hybrid cultivars are grown mainly in fields where weed control is challenging.

The withdrawal of glyphosate would significantly affect the utility of GR crop cultivars. For canola, farmers would likely rely upon alternative HR-trait cultivars such as those resistant to triazine or imidazolinone herbicides. An alternative option would be to use GR cultivars grown in a ‘conventional’ manner (e.g., PRE trifluralin), which may be more expensive depending on the yield advantage, weed spectrum present and herbicides available. Similarly, cotton cultivars with resistance to alternative HR traits such as glufosinate, dicamba and/or 2,4-D may surplant GR cultivars. In the U.S. this trend is already occurring because of the need to control GR weeds [22].

## 4. Bioeconomic Model Simulations: Farming without Glyphosate

Brookes et al. [50] described the global contribution of glyphosate to agriculture and potential adverse economic and environmental impact of restrictions on use (Table 3). With loss of glyphosate, global annual soybean, maize and canola production are predicted to fall by 18.6, 3.1, and 1.5 million tonnes, respectively; herbicide use is expected to increase by 8.2 million kg ai (1.7%) [50]. As stated above, the redundant GR trait in these crops would result in greater reliance on other existing HR traits, such as glufosinate and auxinic resistance, and associated herbicides [22]. The additional cropping area to compensate for lost productivity was estimated at 0.76 million ha (half from land brought into annual crop production), resulting in additional carbon dioxide emissions of 234 billion kg. Two years later, another report examined possible consequences of restrictions on use of glyphosate in seven countries in the Asia-Oceania region [34]. Restriction or ban on glyphosate use is estimated to result in greater use of alternative herbicides as well as manual, mechanical and cultural weed control methods, increasing annual weed control costs by USD 22–30 ha^−1^.

We utilized a bioeconomic simulation model to conduct various scenarios of grain cropping without glyphosate. The model used was RIM, Ryegrass Integrated Management [51,52], to assess weed control (major weed being annual ryegrass), crop productivity and profitability in southern Australia broadacre cropping scenarios with (i.e., control) and without glyphosate. 

### RIM Model: Annual Ryegrass Management in Southern Australia without Glyphosate

Ryegrass Integrated Management (RIM) is a model-based decision support system that allows users to test and compare the long-term (10 year) performance and profitability of various weed control options used in Australian cropping systems. The objective of RIM is not to simulate complex biophysical and environmental mechanisms, but to monitor the impact of production and management practices on weed populations within farming systems dominated by annual grain crops. RIM comprises several components, including the user interface; a visual basic for applications (VBA) framework that provides the interface with various functionalities such as navigation, exporting outputs, etc.; a population dynamic model encompassing several aspects of the annual ryegrass life cycle including germination, plant and seed survival, intra- and interspecific competition, seed production and seed bank persistence; and a rule-based model that links all the components. The original RIM model was released in 2004, with the latest version released in 2013 [51,52].

The RIM model was used to predict average annual gross margins and average residual weed and seed bank densities in a simple (wheat-canola) and diverse (wheat-canola-barley-lupin) no-tillage crop rotation in southern Australia. Simulation results over 10 years for these two crop rotations with (i.e., control) and without knockdown and pre-harvest glyphosate treatments are summarized in Table 4. In the two-year wheat-canola rotation, average annual gross margins for the control and no-glyphosate simulations were AUD $256 and $347 ha^−1^, respectively. Therefore, the profit margin was not reduced in the no-glyphosate scenario. The difference in gross margins largely reflects greater yield potential with earlier sowing. However, average residual (post-harvest) weed density was greater in the no-glyphosate than control simulation (4.6 vs. 0.4 plants m^−2^). Similarly, the average residual seed bank density was greater in the no-glyphosate vs. control simulation (17.0 vs. 2.4 seeds m^−2^, respectively). In the diverse rotation, average annual gross margins for the control and no-glyphosate scenarios were AUD $225 and $303 ha^−1^, respectively. The residual weed densities in the no-glyphosate and control scenario simulations were 5.4 and 0.4 plants m^−2^; residual weed seed banks were 18.1 and 2.3 seeds m^−2^, respectively (Table 4). 

Use of the most effective PRE soil residual herbicide treatments, high crop seeding rate and HWSC only partially compensated for the lack of knockdown and pre-harvest glyphosate applications. For this scenario, synergistic cultural weed control achieved using effective combinations of agronomic practices or tactics will be vitally important to maintain the lowest weed seed bank possible [53]. Nonetheless, the modelling showed that it may be possible to maintain crop yields in southern Australia without glyphosate through early seeding. Mechanical weed control was not employed in these simulations, but their strategic use may augment cultural weed management. The prevalent weed management philosophy of a ‘zero tolerance’ weed seed bank in Australia (e.g., in [54]) may have to be relaxed in favour of the ‘lowest possible’ seed bank unless additional non-herbicidal tactics can be employed to control weed escapes.

## 5. Conclusions and Future Directions

An entire generation of farmers in developed countries, particularly in North and South America and Australia, have known nothing other than glyphosate-based conservation-tillage cropping systems. In general, herbicide alternatives to glyphosate are very limited, less effective and more expensive. Effectively and profitably managing troublesome weeds in major agronomic field crops without glyphosate will be challenging and demand new knowledge and skills to transition successfully. If glyphosate is restricted or banned, loss of additional pesticides such as paraquat, diquat or 2,4-D may soon follow. Therefore, contingency plans should not solely focus on a scenario of farming without glyphosate, but more broadly address farming with restricted herbicide availability. On the positive side, this potential future situation can be viewed as an opportunity for significantly greater adoption of ecologically based weed management tactics, strategies and systems [55]. The global pandemic of multiple-resistant weed populations, demands from grain buyers and restricting social licence to use herbicides in general, all point to reduced herbicide dependency as an imperative future vision and goal for weed management. However, advancing this goal will require viable alternative solutions to manage weeds effectively and profitably, both in the short- and longer-term. 

There are myriad research and development needs for growers and land managers to successfully transition to a limited herbicide world. This back-to-the-future scenario is predicated on minimal or strategic tillage that is especially crucial for soil health (e.g., carbon sequestration) and for soil water conservation in semiarid agroregions. These research and development needs range from short-term practices or tactics such as effective alternative pre-seeding or pre-harvest herbicide options and non-herbicidal tools to longer-term studies that can identify pest (weed, insect, pathogen)-resilient farming systems [56]. Such diverse systems would integrate effective combinations of complementary, synergistic weed control tactics. In southern Australia in particular, research and development will be required to advance alternative non-herbical weed management tools, weed-competitive or earlier-maturing crop cultivars, breeding of cover crops that prematurely terminate early for soil moisture conservation or are terminated by non-chemical methods such as knife-rolling, digital or precision agriculture for site-specific weed management (patch mapping, prescription or real-time weed management tactics), and high biomass, low soil disturbance farming systems (e.g., ‘strip and disc’ system using a stripper header on the combine harvester and seeding into stubble using a disc opener) [57]. 

Transdisciplinary research and development ranging from plant breeding and agronomy to engineering and sociology will be more important than ever in tackling this immense challenge. A good first step is to learn from growers or land managers who have already successfully made this transition and who can provide valuable insight into best management practices, strategies and farming systems for achieving acceptably low weed seed banks with limited pesticides. Criteria for any strategic tillage operation to replace herbicide application would include minimum horsepower and fuel use, cover the field rapidly, shallow soil depth to minimize water loss, crop residue retention on the soil surface for erosion control and maintenance of soil organic carbon.

An even bigger and broader global challenge is how to maintain or increase food and feed production, given population growth projections to 2050 and expected decline in crop productivity because of limited availability of pesticides. What will be the impact of a reduced pesticide world on global greenhouse gas emissions, air, soil and water quality, or land or natural resource management? These broader potential impacts and consequences, both intended and unintended, must be part of the discussions surrounding restricted glyphosate (pesticide) usage. There are always tradeoffs; the short- or long-term benefits and costs to society of every technology must be carefully weighed based on the available evidence. 

## Figures and Tables

**Table 1:** Glyphosate active ingredient use globally (1974–2014) (adapted from Benbrook [17]). Agricultural use comprises 90% of total use. Note: 2014 usage was 825.8 million kg.

Period	Use (Million kg)
1974	3.2
1975–1984	130.5
1985–1994	387.3
1995–2004	1909
2005–2014	6133
Total	8563

**Table 2:** Glyphosate active ingredient use in the United States in 2014, by crop (adapted from Benbrook [17]).

Crop	Use (Million kg)
Soybean	55.7
Maize	31.2
Cotton	7.9
Wheat	7.9
Alfalfa	4.0
Sorghum	1.9
Sugar beet	1.3
Oranges	0.8
Barley	0.5
Canola	0.1
Other	2.1
Total agricultural	113.4
Total agricultural and non-agricultural	125.4

**Table 3:** Predicted percentage change (positive or negative) in crop production with a ban on the use of glyphosate (adapted from Brookes et al. [50]).

Crop	U.S.	Canada	EU	Brazil	South America	Others	World
Rice	0.2	-	0.2	−0.1	−0.6	0	0
Wheat	0.4	0.6	0.1	−0.4	−1.1	0	0.1
Coarse grains (mainly maize)	−2.3	0.8	0.1	−0.8	−1.6	0.2	−0.6
Soybean	−1.9	−5.6	7.5	2.7	−17.1	1.4	−3.7
Canola/rapeseed	−0.1	−5.6	1.7	2.9	1.6	0	−0.7
Other oilseeds	3.3	2.8	2.3	2.7	2.5	1.1	1.4
Palm fruit	6.8	-	3.1	3.6	4.8	0.5	0.7
Sugar crops	0	−0.6	0	−0.2	0	0	−0.1
Other	0.2	0.4	0.1	−0.5	−1.1	0	0

**Table 4:** Ryegrass Integrated Management (RIM) model scenarios (10-year span; southern Australia): simple or diverse no-tillage crop rotations with (control) and without glyphosate: management operations *, average annual gross margin (AUD $) and average annual residual weed and seed bank densities. See Lacoste [52] for model details.

	Wheat-Canola		Wheat-Canola-Barley-Lupin	
	Control	Without glyphosate	Control	Without glyphosate
Operations:				
Time of sowing	Avg. dry	Early dry	Avg. dry	Early dry
Double-knock	Yes	No	Yes	No
PRE herbicides	Wheat: trifluralin	Wheat: Sakura (yr1,5,9), Boxer Gold (yr3,7)	Wheat, barley, lupin: trifluralin	Wheat: Sakura (yr1), trifluralin (yr5,9)
	Canola: triazine	Canola: triazine	Canola: triazine	Barley: Boxer Gold
				Canola: triazine
				Lupin: Sakura
Crop seeding rate	Standard	High	Standard	High
POST herbicides	Canola: triazine + gp A	Canola: triazine + gp A	Canola: triazine + gp A	Canola: triazine + gp A
			Lupin: gp B	Lupin: gp B
Crop-topping	Wheat, yr1	No	Wheat, yr1	Lupin: paraquat
(pre-harvest)				
Swath with spray	Canola, yr2	No	Canola, yr2	No
HWSC: HSD	Yes	Yes	Yes	Yes
Results:				
Gross margin ($ ha^−1^)	256	347	225	303
Weeds (no. m^−2^)	0.4	4.6	0.4	5.4
Weed seeds (no. m^−2^)	2.4	17.0	2.3	18.1

* Avg. dry: ca. 2 weeks later than early dry; double-knock: glyphosate followed by paraquat; triazine: e.g., atrazine; gp A: acetyl-CoA carboxylase inhibitor; Sakura™: pyroxasulfone; Boxer Gold™: prosulfocarb + metolachlor (common names listed for generic herbicides). Control treatments: in addition to double-knock, glyphosate is also used in crop-topping and swath with spray. Abbreviations: gp, group; HSD, Harrington Seed Destructor; HWSC, harvest weed seed control; POST, post-emergence; PRE, pre-emergence; yr, year.
